# Health Care Access Expansions and Use of Veterans Affairs and Other Hospitals by Veterans

**DOI:** 10.1001/jamahealthforum.2022.1409

**Published:** 2022-06-10

**Authors:** Jean Yoon, Kenneth W. Kizer, Michael K. Ong, Yue Zhang, Megan E. Vanneman, Adam Chow, Ciaran S. Phibbs

**Affiliations:** 1 Health Economics Resource Center, VA Palo Alto Health Care System, Menlo Park, California; 2 Sean N. Parker Center for Asthma and Allergy Research, Stanford University, Stanford, California; 3 VA Center for the Study of Healthcare Innovation, Implementation and Policy, Los Angeles, California; 4 Informatics, Decision-Enhancement and Analytic Sciences Center, VA Salt Lake City Health Care System, Salt Lake City, Utah

## Abstract

This cohort study examines changes in the use of Veterans Affairs (VA) and non-VA hospitals by VA enrollees and mortality associated with these policies.

## Introduction

Veterans enrolled in the Veterans Affairs (VA) health care system have access to non-VA care through VA community care programs or other health insurance coverage. Access has increased in recent years because of the expansion of Medicaid programs under the Affordable Care Act and the US Veterans’ Choice Act (VCA), which increased VA-paid community care.^1,2,3^ The association between these policies and utilization and outcomes is unknown. We examined changes in the use of VA and non-VA hospitals by VA enrollees and mortality associated with these policies from January 2012 to December 2017.

## Methods

We conducted a longitudinal study with repeated cross-sections of veterans for a total of 13 million veteran-years in 5 diverse states (Arizona, California, Florida, New York, and Pennsylvania) from 2012 to 2017. We linked VA enrollment records, VA acute hospital records, and all-payer hospital discharge data for a comprehensive set of hospitalization records for VA enrollees. We measured veteran characteristics (age, sex, race and ethnicity [self-reported], marital status, VA enrollment category, and state) and date of death from VA administrative data, the number of hospitalizations by system and payer from VA and state discharge data, and community characteristics (median income, median educational attainment, and unemployment rate) from US Census data. We used enrollees as the unit of analysis in negative binomial models that estimated the number of hospitalizations for each system/payer.^4^ Separate models tested for changes occurring after (1) Medicaid expansion by state and year^5^ and (2) the post-VCA period beginning with 2015, the first full year of implementation. All models adjusted for yearly trend, enrollee and community characteristics, state, and enrollee random effects (eMethods in the Supplement). A similar logistic model was used to estimate death. The analysis was conducted from January 6, 2022, to April 2, 2022. The study was approved by the Stanford University, University of Utah, and Greater Los Angeles VA institutional review boards. Informed consent was waived because of the minimal risk to patients and the fact that the research could not be practicably conducted without the waiver. Statistical analyses were conducted using Stata, version 17 (StataCorp).

## Results

During the 6-year period, the number of VA enrollees in the 5 states grew from 2.2 to 2.4 million. The mean age (61-62 years) and the percentage of men (92%-94%) were similar, while the percentage of enrollees with military service–connected disabilities increased from 24% to 33%. There were fewer total hospitalizations among enrollees over time. The mean number of VA acute hospitalizations declined 14%, whereas the mean number of VA-paid community hospitalizations grew 26% (Figure). Medicare covered more hospitalizations (54%) than any other payer. Medicaid covered only 2% of hospitalizations throughout the study period.

**Figure.**
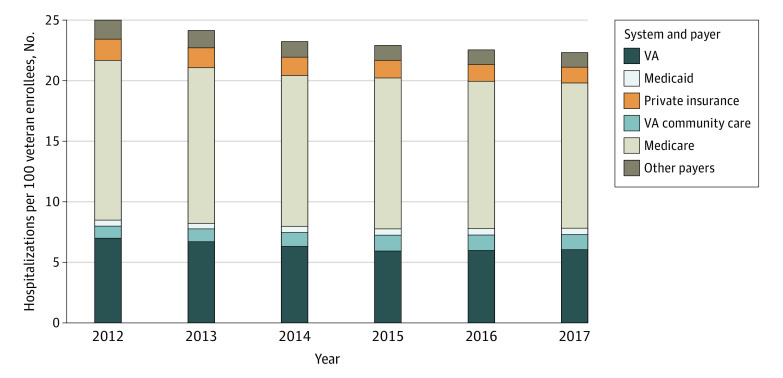
Total Number of Hospitalizations per 100 Veterans Enrolled in the Veterans Affairs (VA) Health Care System by System and Payer From 2012 to 2017

In an adjusted analysis, VA-provided hospitalizations decreased 4% and VA-paid community hospitalizations increased 5% after VCA implementation (Table). Although the number of Medicaid hospitalizations was small, Medicaid expansion was associated with a 19% increase in these hospitalizations and a 2.5% decrease in VA hospitalizations (both *P* < .001). There was no change in mortality following the VCA or Medicaid expansion.

**Table.** Difference in Hospitalization Rates by System and Payer and Mortality Following National Access Policies

Characteristic	Mean difference in outcome rates, % (95% CI)^a^			
	Hospitalizations			Mortality
	VA	VA-paid community	Medicaid	
Post-VCA period	−4.3 (−5.3 to −3.2)	5.0 (2.6 to 7.3)	NA	0.02 (−0.02 to 0.06)
Medicaid expansion	−2.5 (−3.4 to −1.5)	NA	19.3 (15.9 to 22.7)	0.002 (−0.03 to 0.04)

Abbreviations: NA, not applicable; VA, Veterans Affairs; VCA, Veterans’ Choice Act.

^a^ Marginal effects for system and payer were estimated from negative binomial models that predicted the number of hospitalizations that adjusted for the post-VCA period or Medicaid expansion, year, enrollee and community characteristics, state fixed effects, and enrollee random effects. Marginal effects represented the difference in hospitalization rates. A logistic model was used to estimate mortality, and marginal effects from these models represented the difference in probability of death.

## Discussion

To our knowledge, this is the first study since the VCA and Medicaid expansion to examine changes in hospitalizations while using all-payer hospital data. Veterans increased use of community hospitals paid by VA and Medicaid and decreased use of VA hospitals when access to non-VA care expanded. This shift in hospitalizations to the community was not associated with changes in mortality, although other outcomes need to be assessed to understand how changes in hospital utilization were associated with quality of care. While the hospitalizations of most veterans were covered by Medicare, many continued to rely on VA hospitals. Study limitations include an inability to draw causal inferences, lack of data on outpatient care, and lack of generalizability beyond the study states.

The US VA MISSION Act of 2018 further expanded the access of veterans to community care and is expected to amplify the trends observed in this study. Better surgical outcomes were recently documented in VA hospitals compared with the private sector,^6^ so greater privatization of care for veterans may not translate to improved quality and outcomes. Shifting inpatient care to non-VA hospitals poses substantial challenges for care coordination across clinicians and health care systems and requires that outcomes and quality of care be closely monitored.^3^

## Notes

Supplement. eMethods.
