# Supplementary material for: Health Care Access Expansions and Use of Veterans Affairs and Other Hospitals by Veterans
Source: JAMA Health Forum. 2022 Jun 10;3(6):e221409. doi: 10.1001/jamahealthforum.2022.1409 (PMC9187948; doi:10.1001/jamahealthforum.2022.1409)
Supplement: Supplement. — eMethods. [file jamahealthforum-e221409-s001.pdf]

Supplemental Online Content

Yoon J, Kizer KW, Ong MK, et al. Health care access expansions and use of Veterans Affairs and other hospitals by veterans. *JAMA Health Forum*. 2022;3(6):e221409. doi:10.1001/jamahealthforum.2022.1409

eMethods.

This supplemental material has been provided by the authors to give readers additional information about their work.

© 2022 Yoon J et al. *JAMA Health Forum*.

eMethods

Cohort and Data sources

Veterans included in our cohort had to be enrolled in the VA health care system and documented with a record in the VA Assistant Deputy Under Secretary for Health (ADUSH) Enrollment File, have an address with a zip code in one of the 5 study states in order for us to conduct a data linkage with state discharge data, and have complete information for age, gender, and VA enrollment category for a total sample size of 13.5 million Veteran-years.

All VA data was obtained from the VA Corporate Data Warehouse. VA hospitalizations were obtained from VA inpatient utilization data. Non-VA hospitalizations and the primary payer of the hospitalization were obtained from state hospital discharge data. Mortality was measured from the VA Vital Status File.

In regression models we included independent variables for Veterans' individual characteristics obtained from VA Observational Medical Outcomes Partnership (OMOP) Files and community characteristics obtained U.S. Census data. We used a continuous measure of age, gender was categorized as male or female, marital status was categorized as married, divorced/separated/widowed, single, or unknown marital status, and VA enrollment category was grouped by VA-assigned priority for care. Veterans' race/ethnicity was self-reported; categories included Black, Hispanic, Non-Hispanic White, and Other (including Asian American, Pacific Islanders, American Indian and Alaska Native, and unknown race/ethnicity). Veterans with unknown race/ethnicity were included in the cohort since Veteran enrollees using little VA care did not have race/ethnicity reported in VA data. U.S. Census data for median

© 2022 Yoon J et al. *JAMA Health Forum*.

income, highest educational attainment, and unemployment rate was linked to Veterans' zip codes.

Analytic methods

Separate regression models were used to estimate the change in hospitalizations by system/payer associated with (1) Medicaid expansion and (2) the VCA. Models used to estimate the changes associated with Medicaid expansion used a state and year-specific indicator to indicate the time period each state had implemented a Medicaid expansion program. For AZ, CA, and NY, Medicaid expansion was indicated for 2014-2017. For PA, Medicaid expansion was indicated for 2015-2017. FL did not have a Medicaid expansion program in the study period. We also included a continuous measure for year which captured the average underlying time trend in hospitalizations before the policy began. These models estimated VA and Medicaid hospitalizations. Models used to estimate changes associated with the VCA included an indicator for the post-VCA period (2015-2017) for all states since it was implemented nationally in November 2014, and we expected impacts to occur beginning with the first full year of implementation in 2015. We also included a continuous measure for year. In these models, we estimated VA and VA-paid community hospitalizations. We did not include indicators for both policies in the same model because of the overlap in time period for the two policies. In all models we included a fixed effect for state so that we could estimate the change in hospitalizations for each system and payer within each state.

We followed the STROBE guidelines for reporting cohort studies.

© 2022 Yoon J et al. *JAMA Health Forum*.
